# Mitochondrial antioxidants abate SARS-COV-2 pathology in mice

**DOI:** 10.1073/pnas.2321972121

**Published:** 2024-07-15

**Authors:** Joseph W. Guarnieri, Timothy Lie, Yentli E. Soto Albrecht, Peter Hewin, Kellie A. Jurado, Gabrielle A. Widjaja, Yi Zhu, Meagan J. McManus, Todd J. Kilbaugh, Kelsey Keith, Prasanth Potluri, Deanne Taylor, Alessia Angelin, Deborah G. Murdock, Douglas C. Wallace

**Affiliations:** ^a^The Center for Mitochondrial and Epigenomic Medicine, The Children's Hospital of Philadelphia, Philadelphia, PA 19104; ^b^University of Pennsylvania, Philadelphia, PA 19104; ^c^Department of Microbiology, Perelman School of Medicine, University of Pennsylvania, Philadelphia, PA 19104; ^d^Department of Anesthesiology and Critical Care, Children's Hospital of Philadelphia, Philadelphia, PA 19104; ^e^Department of Biomedical and Health Informatics, The Children's Hospital of Philadelphia, Philadelphia, PA 19104; ^f^Division of Human Genetics, Department of Pediatrics, Perelman School of Medicine, University of Pennsylvania, Philadelphia, PA 19104

**Keywords:** mitochondria, SARS-CoV-2, antioxidant therapy, mCAT, EUK8

## Abstract

Severe Acute Respiratory Syndrome Coronavirus 2 (SARS-CoV-2) infection inhibits mitochondrial oxidative phosphorylation (OXPHOS) and elevates mitochondrial reactive oxygen species (ROS, mROS) which activates hypoxia-inducible factor-1alpha (HIF-1α), shifting metabolism toward glycolysis to drive viral biogenesis but also causing the release of mitochondrial DNA (mtDNA) and activation of innate immunity. To determine whether mitochondrially targeted antioxidants could mitigate these viral effects, we challenged mice expressing human angiotensin-converting enzyme 2 (ACE2) with SARS-CoV-2 and intervened using transgenic and pharmacological mitochondrially targeted catalytic antioxidants. Transgenic expression of mitochondrially targeted catalase (mCAT) or systemic treatment with EUK8 decreased weight loss, clinical severity, and circulating levels of mtDNA; as well as reduced lung levels of HIF-1α, viral proteins, and inflammatory cytokines. RNA-sequencing of infected lungs revealed that mCAT and Eukarion 8 (EUK8) up-regulated OXPHOS gene expression and down-regulated HIF-1α and its target genes as well as innate immune gene expression. These data demonstrate that SARS-CoV-2 pathology can be mitigated by catalytically reducing mROS, potentially providing a unique host-directed pharmacological therapy for COVID-19 which is not subject to viral mutational resistance.

## Significance

Severe Acute Respiratory Syndrome Coronavirus 2 (SARS-CoV-2) continues to evolve its Spike (S) protein sequence to avoid immunization constraints. To develop a more durable intervention to combat COVID-19, we determined that SARS-CoV-2 inhibits mitochondrial oxidative phosphorylation (OXPHOS) to increase mitochondrial reactive oxygen species (mROS) production which activates hypoxia-inducible factor-1alpha (HIF-1α) to shift metabolism from OXPHOS to glycolysis, thus redirecting substrates toward viral biogenesis. To counteract this viral strategy, we increased the mitochondrial antioxidant capacity of mice by mitochondrially targeted catalase or EUK8 and demonstrated that these interventions significantly reduced the pathology of SARS-CoV-2 infection. This strategy would not be subject to SARS-CoV-2 S gene mutational resistance and might mitigate the pathology of other viruses.

COVID-19 is caused by the single-stranded RNA virus, Severe Acute Respiratory Syndrome Coronavirus 2 (SARS-CoV-2). SARS-CoV-2 causes mitochondrial dysfunction characterized by altered mitochondrial morphology, oxidative phosphorylation (OXPHOS) inhibition, elevated mitochondrial reactive oxygen species (mROS), and mitochondrial-induced apoptosis. Inhibited OXPHOS and associated increased mROS activate hypoxia-inducible factor-1alpha (HIF-1α), which induces HIF-1α target genes and shifts gene expression from OXPHOS toward glycolysis thus redirecting substrates to viral replication (1–17). OXPHOS dysfunction and increased mROS have been linked to the release of mtDNA into the cytoplasm and activation of the innate immune system. Cytosolic mtDNA binds to the mitochondrially bound NOD-LRR-pyrin domain-containing protein 3 (NLRP3)-containing inflammasome (NLRP3-I), which activates caspase-1 (CASP1) to cleave pro-interleukin 1 beta (proIL-1β) to IL-1β triggering inflammation along with activating gasdermin D (GSDMD) initiating pyroptosis (2, 18, 19). Cytosolic mtDNA also interacts with cyclic guanosine monophosphate-adenosine monophosphate (GMP–AMP) synthase (cGAS), which acts through cyclic GMP–AMP to activate stimulator of interferon genes (STING) and the Type I interferon response. Cytoplasmic mtDNA also binds to and activates toll-like receptor 9 (TLR9), which acts through its adaptor molecule myeloid differentiation factor 88 (MyD88). Activated TLR9-MyD88 and IL-1R1 induce nuclear factor kappa B (NF-κB) while cGAS-STING activates interferon regulatory factors (IRFs), resulting in the transcription of pro-inflammatory cytokine genes (20–22).

ROS scavengers N-acetylcysteine and MitoQ can impair SARS-CoV-2 replication and IL-1β mRNA expression in infected peripheral blood monocytes (5). This may be mediated by the viroporin proteins E and Orf3a whose expression in HEK293T cells increases Ca^++^ influx into the cell and mitochondria, elevates mROS production, and releases fragmented mtDNA through the mitochondrial permeability transition pore (mtPTP) to bind and activate the NLRP3-I. In cultured cells, these processes can be inhibited by the mitochondrially targeted catalytic antioxidants, MnTBAP, and mitochondrially targeted catalase (mCAT), or the mtPTP-specific inhibitor, NIM811 (2).

In SARS-CoV-2 infected mice, expressing the viral receptor human angiotensin-converting enzyme 2 (hACE2) transcribed from the human keratin 18 (K18) promoter (K18-hACE2) (23), mitochondrial OXPHOS proteins were downregulated in the heart, lung, kidney, and spleen (24). We therefore hypothesized that we might disrupt SARS-CoV-2 pathogenicity in K18-hACE2 mice by reducing mROS production. To effectively remove mROS, we employed the mitochondrially targeted catalytic antioxidants, the mCAT transgene and the drug, EUK8. Systemic expression of mCAT in the mouse reduces mROS and mtDNA damage and extends mouse lifespan (25, 26). Treatment of mice with partially impaired mitochondrial antioxidant defenses with EUK8 mitigates the neurological phenotype and also extends lifespan (27). Both mCAT expression and EUK8 treatment reversed OXPHOS inhibition, reduced HIF-1α and glycolytic gene expression, reduced mtDNA release mitigating innate immune activation, and impaired SARS-CoV-2 biogenesis.

## Results

### Systemic Expression of mCAT in SARS-CoV-2 Infected hACE2 Mice Reverses OXPHOS Defect, Destabilizes HIF-1α, and Mitigates the Virally Induced Immune Activation.

We first employed C57BL/6J K18-hACE2 and C57BL/6J mCAT mice to create mice that were heterozygous for K18-hACE2 (hACE2) and homozygous for mCAT (hACE2-mCAT). We then confirmed that the human catalase was expressed in the lungs by immunohistochemistry (IHC) (*SI Appendix*, Fig. S1). Additionally, when challenged with LPS, hACE2-mCAT mice had reduced lung lipid peroxidation levels assessed by malondialdehyde (MDA) assay, compared to hACE2 controls (*SI Appendix*, Fig. S2).

**Fig. 1.**
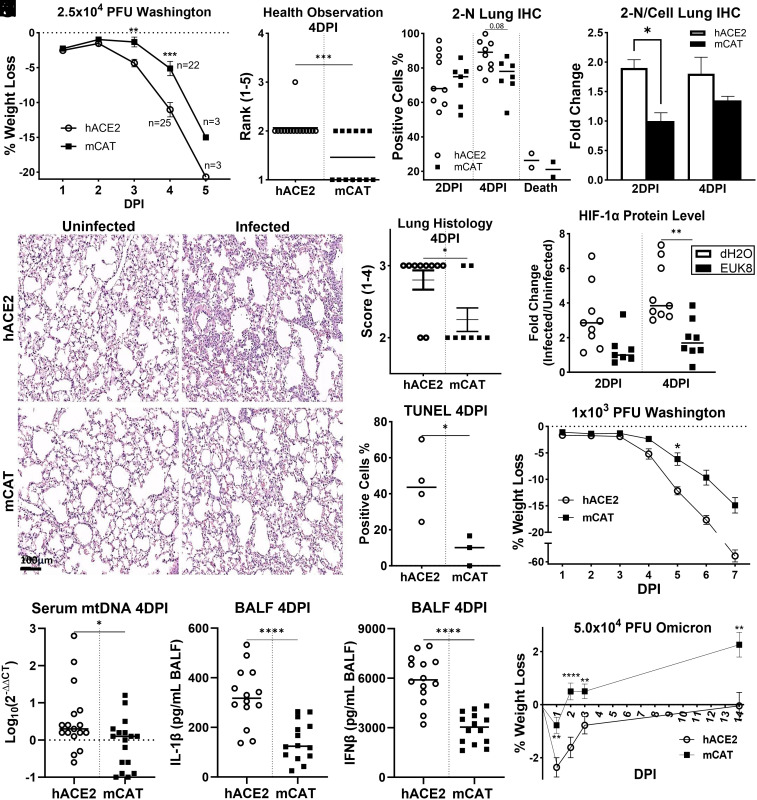
Systemic expression of mCAT in SARS-CoV-2 infected hACE2 mice decreases disease severity. (*A–K*) Mice were infected intranasally with 2.5 × 10^4^ PFU WA1, across three independent experiments (hACE2 versus hACE2-mCAT (mCAT), 2 DPI n = 34/22; 3 to 4 DPI 25/22; 5 to 6 DPI 3/3; mock-infected 11/12): (*A*) weight loss, (*B*) health status scoring of infected mice, (*C*) high-accuracy large-scale object (HALO) analysis of the percentage of 2-N positive stained cells, and (*D*) intensity of 2-N staining in mouse lungs collected at 2, 4, and 5 to 6 DPI across two independent experiments (hACE2 versus hACE2-mCAT, 2 DPI n = 9/7; 4 DPI 10/8; 5 to 6 DPI 2/2; mock-infected 5/6). (*E*) Hematoxylin and eosin (H&E)-stained lung samples across two independent experiments (hACE2 versus hACE2-mCAT, 4 DPI 10/8; mock-infected 5/6). (*F*) Pathological scoring of lung histology staining (*E*) at 4 DPI. (*G*) HALO analysis of the percentage of terminal deoxynucleotidyl transferase dUTP nick end labeling (TUNEL)-stained positive lung cells at 4 DPI (hACE2 versus hACE2-mCAT, 4 DPI 4/3; mock-infected 3/3). (*H*) HALO analysis of the percentage of HIF-1α-stained positive cells, across two independent experiments (hACE2 versus mCAT, 2 DPI n = 9/7; 4 DPI 10/8; 5 to 6 DPI 2/2; mock-infected 5/6); representative images of anti-HIF-1α antibody stained infected lungs stained presented in *SI Appendix*, Fig. S3. (*I*) mCAT effects on relative mtDNA serum levels of infected versus uninfected mice at 4 DPI determined by RT-PCR with a probe specific for MT-ND5 across two independent experiments (hACE2 versus hACE2-mCAT, 4 DPI 17/17; mock-infected 4/4). DNA was isolated from equal amounts of protein. (*J*) mCAT effects on relative IL-1β levels determined in BALF by ELISA in mice 4 DPI across two independent experiments (hACE2 versus hACE2-mCAT, 4 DPI 14/14; mock-infected 4/4). (*K*) mCAT effects on relative INFβ levels determined as in *J*. (*L*) Weight loss of mice nasally infected with 1.0 × 10^4^ PFU WA1 (hACE2 versus hACE2-mCAT 12/8). (*M*) Weight loss of mice nasally infected with 5.0 × 10^5^ PFU Omicron BA.1 (hACE2 versus hACE2-mCAT, 3 DPI n = 16/23, 14 DPI = 6/8, mock-infected 3/3). Error bars represent STD, and statistically significant data are indicated with asterisks (*).

We infected hACE2 and hACE2-mCAT mice intranasally with 2.5 × 10^4^ plaque-forming units (PFU) of the Washington-variant (WA1) and determined whether mCAT expression could ameliorate viral pathology. Compared to hACE2 controls, hACE2-mCAT mice displayed decreased weight loss throughout infection (Fig. 1*A*) and improved clinical score at 4 days post-infection (DPI) (Fig. 1*B*). This was associated with decreased SARS-CoV-2 nucleocapsid protein (2-N) in the lungs at 2 DPI (Fig. 1 *C* and *D*). At 4 DPI, the SARS-CoV-2-infected hACE2-mCAT mice had histologically assessed increased airway space and decreased multifocal viral foci relative to the hACE2-infected mice (Fig. 1 *E* and *F*). hACE2-mCAT mice also had decreased apoptosis assessed by TUNEL staining at 4 DPI (Fig. 1*G*). SARS-CoV-2 infection stabilized lung HIF-1α resulting in its accumulation at 2 and 4 DPI, and systemic expression of mCAT significantly reduced HIF-1α levels by 4 DPI (Fig. 1*H* and *SI Appendix*, Fig. S3). In cultured cells, mROS initiates the release of mtDNA into the cytoplasm to activate the inflammasome stimulating the maturation of IL-1β (2). This phenomenon was confirmed in SARS-CoV-2 infected mice by showing that mtDNA is released into their serum and that mCAT significantly reduced serum mtDNA levels (Fig. 1*I*). mCAT also dramatically reduced IL-1β (Fig. 1*J*) and IFN-beta (IFN-β) levels (Fig. 1*K*) in bronchoalveolar lavage fluid (BALF) of infected hACE2 mice.

To confirm that these observations were not specific to the SARS-CoV-2 WA1 inoculum or the WA1 strain, we infected hACE2 mice with and without mCAT at a lower WA1 PFU and with the Omicron strain. In both cases, hACE2-mCAT protected the mice from viral-induced weight loss: 1.0 × 10^3^ PFU for WA1 (Fig. 1*L*) or 5.0 × 10^4^ PFU for Omicron BA.1 (Fig. 1*M*).

#### Effects of mCAT expression in SARS-CoV-2 infected lungs determined by RNA-sequencing (RNAseq) confirms the induction of OXPHOS and reduction in HIF-1α and inflammatory gene expression.

**Fig. 2.**
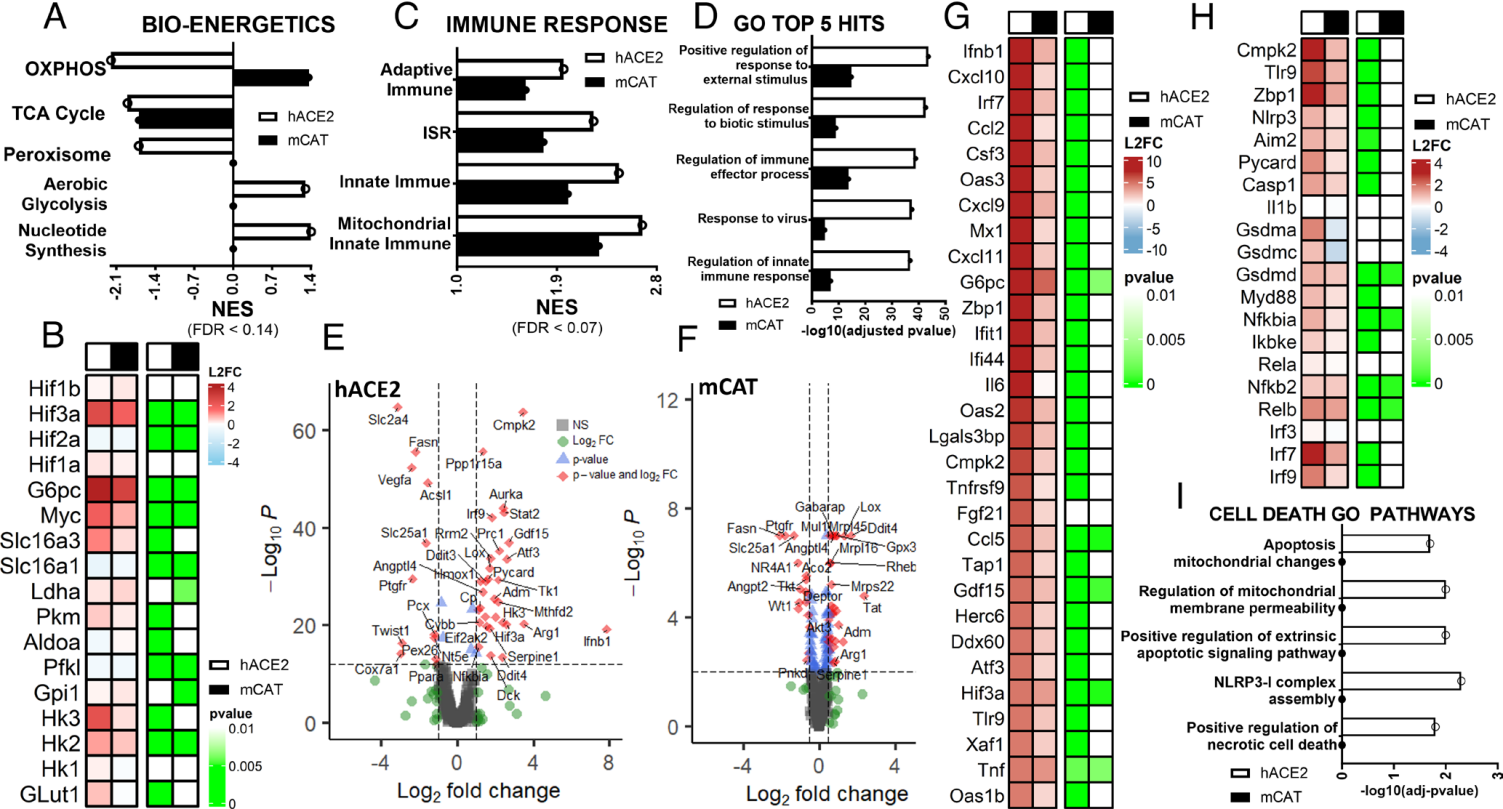
RNAseq analysis of lung mRNAs in SARS-CoV-2 infected hACE2 versus hACE2-mCAT mice revealed that mCAT dampened virus induced metabolic reprogramming and innate immune activation. Analysis of RNA transcripts from mouse lung assessed in five SARS-CoV-2 hACE2-infected mice, five hACE2-mCAT infected mice, three uninfected hACE2 mice, and three uninfected hACE2-mCAT mice collected at 4 DPI. Bar plots for statistically significant changes (FDR < 0.14). (*A*) Effect of mCAT expression showing striking reversal of viral OXPHOS inhibition (1) together with partial reversal of viral inhibition of TCA and peroxisomal gene suppression, determined by GSEA for infected versus uninfected lung samples, ranked by nominal enrichment score (NES) (29). (*B*) Heat map representation of mCAT effects on SARS-CoV-2 infected hACE2 and hACE2-mCAT mice on lung HIF and glycolytic mRNA levels, log-2 fold (L2FC). (*C*) Effect of mCAT expression on lung innate immune gene expression. (*D*) Gene ontology (GO) pathway analysis of RNA transcripts demonstrating the striking reversal of viral innate immune gene induction by mCAT expression. (*E*) Volcano plot of RNA transcript levels of hACE2-infected versus uninfected lung samples. (*F*) Volcano plot of RNA transcript levels of hACE2-mCAT infected versus uninfected lung samples. (*G*) Heat map representation of mCAT effects on SARS-CoV-2 infected hACE2 and hACE2-mCAT mice on innate immune and integrated stress response (ISR) mRNA levels, L2FC. (*H*) Heat map representations of mCAT effects SARS-CoV-2 infected hACE2 and hACE2-mCAT mice on selected mitochondrial, NF-κB, and interferon mRNA levels, L2FC. (*I*) GO pathway analysis of RNA transcripts demonstrating the striking mCAT inhibition of cell death pathways. An O on a line indicates that the difference between with versus without treatment did not change sufficiently to reach FDR < 0.25 or −log10(adj-*P* value) >1.3.

**Fig. 3.**
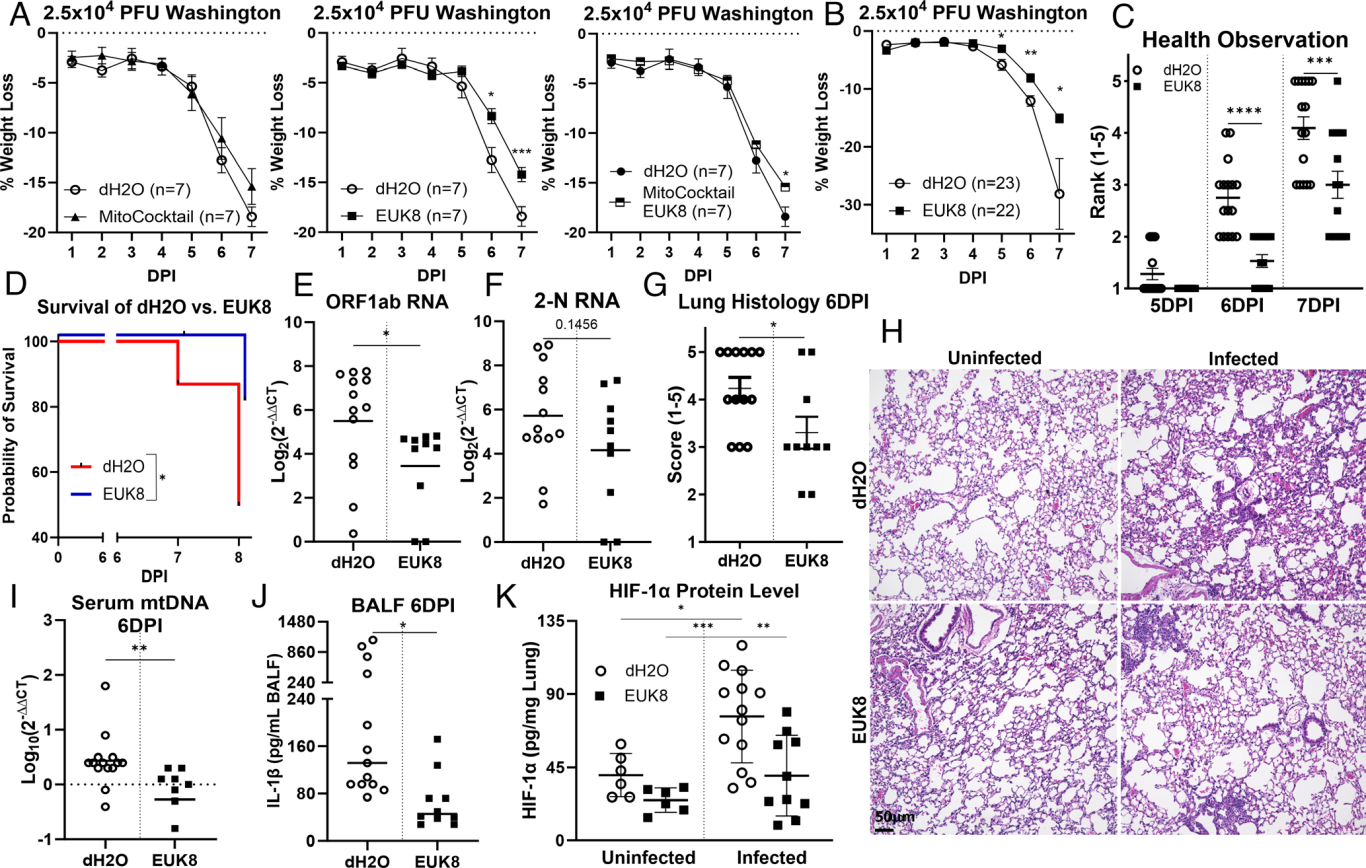
Systemic expression of mitochondrial cocktail (MitoCocktail) and EUK8 on SARS-CoV-2 infected hACE2 mice decreases disease severity. (*A–K*) hACE2 mice were infected intranasally with 2.5 × 10^4^ PFU of the WA1. (*A–D*) dH_2_O/EUK8/MitoCocktail/MitoCocktail+EUK8, 23/22/7/7 infected mice, 6/6/3/3 mock-infected mice, dH_2_O versus EUK8 experiments were assessed across two independent experiments: (*A* and *B*) Weight loss. (*C*) pathological scoring 1 to 5. (*D*) Survival analysis. (*E* and *F*) SARS-CoV-2 ORF1ab and viral N structural protein (2-N) mRNA levels in lungs 6 DPI determined by RT-PCR, dH_2_O/EUK8, 13/10; mock-infected 6/6, error bars represent STD and statistically significant data is indicated with asterisks (*): (*E*) ORF1ab, (*F*) 2-N. (*G*) Quantification of lung histopathology assessed by H&E staining at 6 DPI, samples collected across two independent experiments. (*H*) Representative H&E-stained lung samples of vehicle and EUK8-treated hACE2 mice at 6 DPI. (*I*) Serum levels of mtDNA determined at 6 DPI DNA by RT-PCR with an MT-ND5 probe normalized to serum volume. (*J*) IL-1β levels determined by ELISA in BALF at 6 DPI, IL-1β was undetectable in uninfected mice. (*K*) Lung HIF-1α protein levels determined by ELISA at 6 DPI.

To investigate whether mCAT expression mitigates SARS-CoV-2-induced metabolic and innate immune alterations, we performed RNAseq on the nuclear DNA (nDNA) transcripts from lung samples collected from infected hACE2 and hACE2-mCAT mice at 4 DPI and then calculated the relative expression levels of host genes. mCAT expression reversed the viral inhibition of OXPHOS and partially restored the viral inhibition of the TCA and peroxisomal gene expression (Fig. 2*A*) (28). Viral stabilization of HIF-1α in hACE2-infected lungs was associated with the induction of *Hif-3α* and *Myc*, multiple glycolytic gene mRNAs, and the plasma membrane pyruvate and lactate carrier *Slc16a3*. mCAT expression reversed the induction of glycolytic genes resulting in the downregulation of *Myc, G6pc, Pkm, Hk3, Glut1,* and the *Slc16a3* carrier (Fig. 3*B*).

mCAT also markedly reduced SARS-CoV-2-infected mouse lung adaptive and innate immune system activation and the activation of the integrated stress response (ISR) (Fig. 2*C*), with Gene Ontology (GO) pathway analysis confirming that mCAT strongly suppressed viral activation of immune function (Fig. 2*D*). Volcano plots (Fig. 2 *E* and *F*) and heatmaps (Fig. 2 *G* and *H*) revealed that SARS-CoV-2 induced expression of an extensive array of inflammatory genes and that mCAT reduced the expression of innate immune genes *Ifnb1*, *Ccl2*, *Ccl5*, *Cxcl9*, *Cxcl10*, *Cxcl11*, *IL6*, *Oas1b*, *Oas2, Oas3,* and *Tlr9* and *Xaf1* (Fig. 2 *G* and *H*) plus *Cmpk2*, which encodes cytidine/uridine monophosphate kinase 2 (CMPK2), the rate-limiting enzyme of mtDNA replication (18, 19); *Zbp1* which encodes Z-DNA binding protein 1 (ZBP1), which binds mtDNA, double-stranded RNA, or telomere RNA to activate the MAVS (mitochondrial anti-viral signaling) protein (30); the Gasdermins (*Gsdm a, c, d*) essential for pyroptosis; as well as genes associated with NF-κB, IRFs, and PANoptosis (28, 31) (Fig. 2 *G* and *H*). mCAT also mitigated the SARS-CoV-2 induced expression of the mitochondrial ISR pathway genes including fibroblast growth factor 21 and growth differentiation factor 15 (Fig. 2*G*). Finally, GO pathway analysis confirmed that mCAT mitigated the viral induction of apoptosis, necrosis, and associated cell death pathways genes (Fig. 2*I*).

Thus, mCAT up-regulates lung mitochondrial bioenergetic mRNAs and down-regulates glycolytic genes while reducing mRNAs for the inflammasome (*Cmpk2*, *Aim2*, *Nlrp3*, *Pycard*, and *Casp1*), interferon (*Tlr9*, *Irf7*, and *Irf9),* NF-κB (*Myd88*, *Nfkb2*, *Nfbkia*, and *Relb)*, and cell death (*Zbp1* and *Gsdmd*) pathways.

### Treatment of SARS-CoV-2 Infected hACE2 Mice with Mitochondrially Targeted Catalytic Antioxidant EUK8 Reverses OXPHOS Defect, Destabilizes HIF-1α, and Mitigates Virally Induced Immune Activation.

To clarify the physiological basis of the mCAT benefits for SARS-CoV-2 infection of hACE2 mice, we treated mice with a MitoCocktail to enhance OXPHOS function and the mitochondrially targeted catalytic antioxidant, EUK8, to remove mROS (27, 32–34). The MitoCocktail included the vasodilator (L-arginine), mitochondrial enzyme cofactors (Thiamin, Riboflavin, Niacin, Biotin, Folic-acid, and L-carnitine), antioxidants (α-lipoic acid, Vitamin-C, and Coenzyme Q10), and a mitochondrial substrate (β-hydroxybutyrate) (35–37).

hACE2 mice were peritoneally injected with the MitoCocktail, EUK8, or MitoCocktail plus EUK8 one d before infection with 2.5 × 10^4^ PFU of SARS-CoV-2 WA1. The MitoCocktail-treated mice showed less weight loss than dH_2_O vehicle-treated mice, though not significantly (Fig. 3*A*).

Treatment with EUK8 reduced lung lipid peroxidation levels assessed by MDA assay when challenged with LPS, compared to dH_2_O vehicle-treated mice (*SI Appendix*, Fig. S2). Like mCAT-expression, EUK8 significantly protected the SARS-CoV-2-infected hACE2 mice from weight loss at 5, 6, and 7 DPI (Fig. 3 *A* and *B*). EUK8-treated mice also exhibited a striking protection from SARS-CoV-2 pathogenesis in clinical score at 6 and 7 DPI (Fig. 3*C*) and extended survival (Fig. 3*D*).

The remarkable beneficial effects of EUK8 for hACE2-infected mice correlated with a significant reduction in lung viral protein, Orf1ab (Fig. 3*E*) in association with an apparent reduction in viral N protein (Fig. 3*F*) and a significant reduction in the lung pathology (Fig. 3 *G* and *H*). The EUK8-treated hACE2 mice also had reduced serum mtDNA (Fig. 3*I*), BALF IL-1β (Fig. 3*J*), and lung HIF-1α levels (Fig. 3*K*). Hence, reduction in mROS is a potent inhibitor of SARS-CoV-2 pathogenicity and more effective than stimulation of OXPHOS.

#### Effects of EUK8 treatment on SARS-CoV-2-infected lungs determined by RNAseq recapitulates the induction of OXPHOS and reduction in HIF-1α and inflammatory gene expression.

**Fig. 4.**
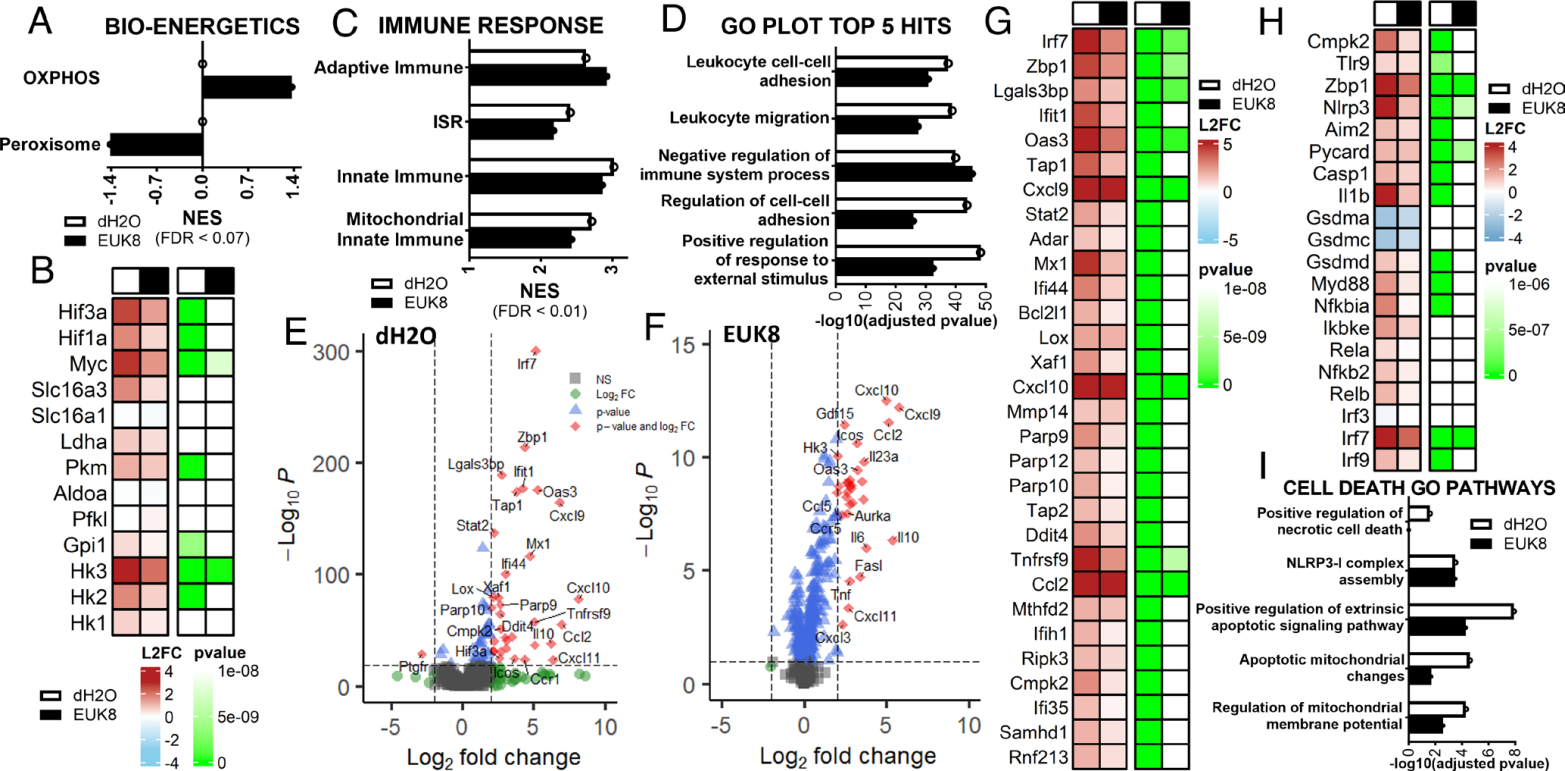
RNAseq analysis of lung mRNAs of SARS-CoV-2 infected hACE2 mice versus EUK8-treated hACE2 mice dampened viral induced metabolic reprogramming and innate immune activation. Analysis of RNA transcripts from mouse lung assessed in five SARS-CoV-2 hACE infected mice, five hACE2-EUK8-treated mice, three uninfected hACE2 mice, and three uninfected hACE2-EUK8 mice collected at 4 DPI. Bar plots for statistically significant changes (FDR < 0.15). (*A*) Effect of EUK8 on lung transcripts showing the striking reversal of viral OXPHOS (1) and peroxisome gene expression determined by GSEA for infected versus uninfected lung samples, ranked by NES, nominal enrichment score (29). (*B*) Heat map altered HIF and glycolytic mRNA levels in SARS-CoV-2 infected hACE2 and EUK8-treated hACE2 mouse lungs, log-2 fold (L2FC). (*C*) Effect of EUK8 treatment on innate immune gene expression. (*D*) GO pathway analysis of RNA transcripts demonstrating the mitigation of viral innate immune gene induction by EUK8 treatment. (*E*) Volcano plot of RNA transcript levels of hACE2-infected versus uninfected lung samples. (*F*) Volcano plot of RNA transcript levels of EUK8-treated hACE2-infected versus uninfected lung samples. (*G*) Heat map representation of SARS-CoV-2-infected hACE2 versus EUK8-treated infected hACE2 mice on innate immune and ISR mRNA levels, L2FC. (*H*) Heat map representation of SARS-CoV-2-infected hACE2 and EUK8-treated hACE2 mice of selected mitochondrial, NF-κB, and interferon mRNA levels, L2FC. (*I*) GO pathway analysis of RNA transcripts demonstrating the EUK8 inhibition of cell death pathways. An O on a line indicates that the difference between with versus without treatment did not change sufficiently to reach FDR < 0.25 or −log10(adj-*P* value) >1.3.

The metabolic and immune effects of EUK8 treatment on the lungs of SARS-CoV-2-infected mice was analyzed by RNAseq of the nDNA transcripts at 4 DPI. EUK8 reversed the inhibition of OXPHOS (Fig. 4*A*) as well as reduced the expression of *Hif-1α, Hif-3α,* and *Myc* resulting in the downregulation of the glycolytic genes *Ldha, Pkm, Gpi1, Hk1*, and *Hk2* plus *Slc16a3* (Fig. 4*B*).

**Fig. 5.**
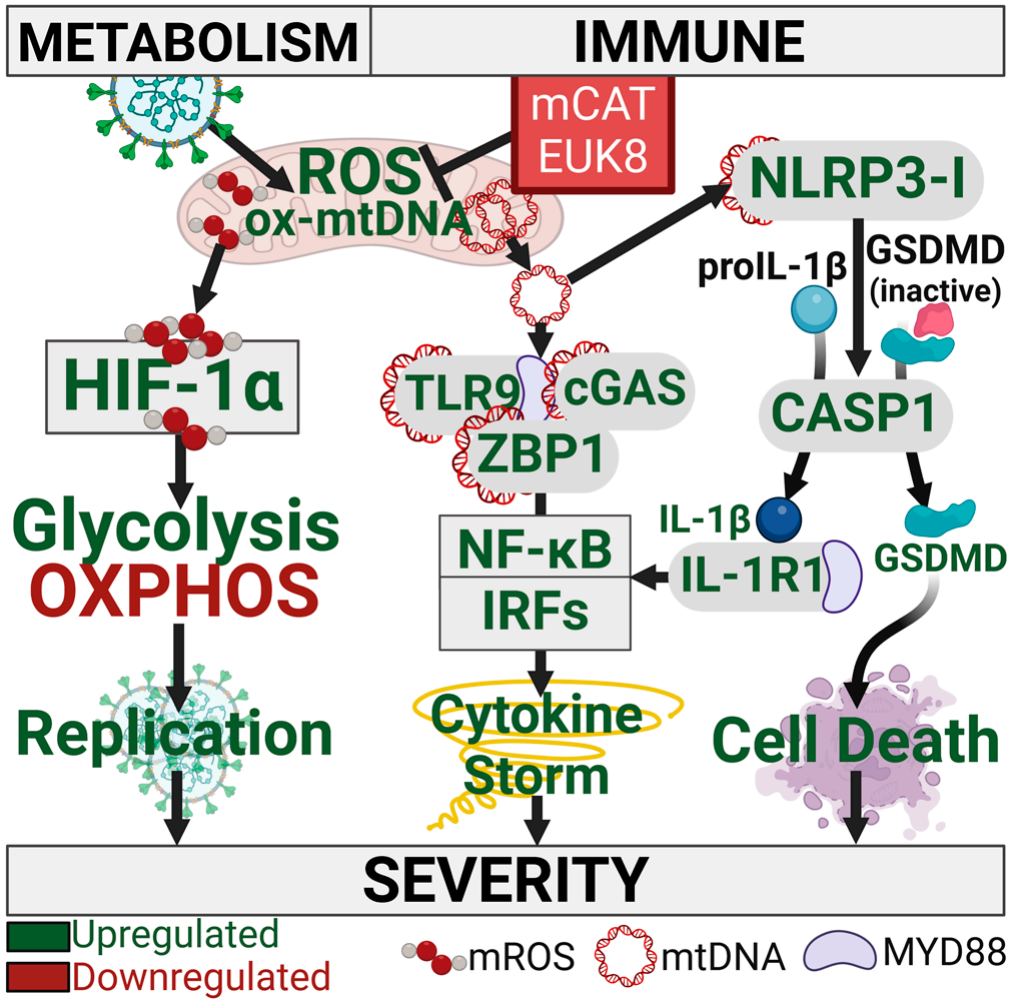
Proposed mitochondrial bioenergetic pathophysiology of SARS-CoV-2 infection and its mitigation by the mCAT transgene and EUK8 treatment. SARS-CoV-2 inhibits mitochondrial OXPHOS at both the protein and transcriptional levels 1) and floods the cell and mitochondria with Ca^++^ via the viroporins E and Orf3a 2) resulting in increased mitochondrial ROS production. *Left* Arm: The increased mROS stabilizes and activates HIF-1α which up-regulates glycolysis and down-regulates OXPHOS redirecting substrates from oxidative energy production to provide substates for viral propagation. *Middle* and *Right* Arm: Increased mROS and mitochondrial Ca^++^ activated the mtPTP to release fragmented and oxidized mtDNA into the cytosol. *Middle* Arm: cytosolic mtDNA interacts with TLR9, cGAS of the cGAS-STING pathway, and ZBP1 which interacts with MAVS molecule to activate the NFκB and interferon inflammation pathways. *Right* Arm: Oxidized mtDNA fragments interact with the NLRP3 inflammasome to activate CASP1 which processed pro-IL-1β to IL-1β which is secreted and activates the inflammation pathways. CASP1 also processes the GSDMD precursor to activated GSDMD which initiates pyroptosis and cell death.

The viral activation of the adaptive and innate immune system and the ISR genes were also reduced by EUK8 (Fig. 4*C*), with immune function being among the most significantly mitigated pathways by EUK8 treatment by GO pathway analysis (Fig. 4*D*). Volcano plots confirmed that expression of critical innate immune genes induced by SARS-CoV-2 infection were downregulated by EUK8 (Fig. 4 *E* and *F*). These included *Zbp1, Irf7, Oas3, Parp 10*, and *12*, *Cxcl 9* and *10*, *Stat2*, and others (Fig. 5 *G* and *H*). This was accompanied by EUK8 counteracting the SARS-CoV-2 induction of NF-κB and cell death pathways associated with *Cmpk2; Casp1; Gsdmd; Tlr9;* and multiple interferon gene expression (Fig. 4 *G* and *H*). EUK8 also reduced the transcripts associated with the apoptosis and necrosis pathways summarized by GO analysis (Fig. 4*I*).

Hence, pharmacological treatment of SARS-CoV-2 infected mice with catalytic antioxidant EUK8 had the same beneficial effects as mCAT. Both catalytic antioxidants up-regulated OXPHOS, down-regulated HIF-1α induced glycolysis, and counteracted innate immune induced inflammation.

## Discussion

We have found that the severity of SARS-CoV-2 infection in mice can be dramatically reduced by the catalytic reduction of mROS through the action of the mCAT transgene, or treatment with the mitochondrially targeted catalytic antioxidant drug, EUK8. The MitoCocktail which should enhance OXPHOS function was less effective at limiting SARS-CoV-2 pathogenicity. Hence, reduction of mROS is the superior strategy for mitigating SARS-CoV-2 pathogenicity.

SARS-CoV-2 infection of the hACE2 receptor expressing mouse lung resulted in the downregulation of lung nDNA coded OXPHOS genes and the upregulation of glycolysis genes in association with stabilization of HIF-1α and induction of *Hif-1α*, *Hif-3α*, and *Myc*. The systemic expression of mCAT or treatment with EUK8 reversed the viral transcriptional inhibition of OXPHOS, blocked the stabilization of HIF-1α, and suppressed expression of *Hif-1α*, *Hif-3α*, and *Myc* thus reducing glycolytic gene expression (Fig. 5). The mtDNA transcripts were not analyzed (16, 38) since SARS-CoV-2 microRNA-2392 modulation of human mtDNA transcription may not be conserved in mouse (1, 28).

mCAT and EUK8 also markedly impaired SARS-CoV-2 induction of the lung innate immune system. This was the result of inhibiting mtDNA release through the mtPTP which activates the inflammasome, the cGAS-STING interferon pathway, TLR9, and the genes associated with apoptosis and PANapoptosis (Fig. 5) (28).

Thus, mitochondrially targeted catalytic antioxidants provide a powerful anti-viral alternative to the current reliance on vaccination against the SARS-CoV-2 Spike (S) protein. Unlike viral protein immunization which selects for viral mutations to minimize S antigenicity, treatment with mitochondrial catalytic antioxidants renders the host metabolism unfavorable for viral propagation making viral mutations less likely to engender resistance. Thus, mitochondrially targeted catalytic antioxidants provide a promising approach to treating SARS-CoV-2, which might be generalizable to other viral infections.

## Materials and Methods

### Study Design.

Mice expressing the SARS-CoV-2 human ACE2 receptor were bred with mice harboring the mCAT catalytic antioxidant mCAT transgene or treated with a MitoCocktail or with the mitochondrially targeted catalytic antioxidant drug EUK8 and infected with SARS-CoV-2. The effects of the antioxidants on the virus infection were then monitored based on weight loss, pathological score, circulating mtDNA and inflammatory cytokines, and survival.

### Limitations of the Study.

This study used the K18-hACE2 mouse which is subject to neurological infection by SARS-CoV-2.

### Generation of Viral Stocks and Mouse Infection.

SARS-CoV-2 Washington WA.1 or Omicron BA.1 variants were propagated in transmembrane serine protease 2 (TMPRSS2) expressing Vero cells. The TMPRSS2 Vero cells were grown in Dulbecco's modified Eagle's medium (DMEM) supplemented with 10% fetal bovine serum. The virus was sequenced after propagation and found to match the input strain.

Twelve-week-old mice were randomly assigned to one of two treatment groups: mock infected and infected. Mice were anesthetized with ketamine–xylazine and infected with 2.5 × 10^4^ PFU's of the SARS-CoV-2 Washington WA.1 or 5.0 × 10^5^ PFU's Omicron BA.1 variants intranasally in 40 μL DMEM.

### Infection Models and Sample Collection.

#### Mice.

Transgenic mice harboring the hACE2 expressed from the human K18 promoter (K18-hACE2) (23) were purchased from the Jackson Laboratory [B6.Cg-Tg(K18-ACE2)2Prlmn/J, Strain #:034860]. The hACE2 transgene is expressed in epithelia, including airway epithelia (23).

The mCAT transgenic mice involve the human catalase (*CAT*) cDNA lacking the peroxisomal targeting sequence fused to 25 amino acids of the mitochondrial targeting sequence from the ornithine transcarbamylase (*OTC*) gene transcribed from CMV enhancer/chicken beta-actin promoter and were purchased from the Jackson Laboratory [B6.Cg-Tg(CAG-OTC/CAT)4033Prab/J, Strain #:016197] (25, 26). To generate mice heterozygous for hACE2 and homozygous for mCAT (hACE2-mCAT), we prepared mice homozygous to mCAT but lacking hACE2 and mice homozygous for both mCAT and hACE2. We then crossed these two strains and studied the F1 progeny.

#### EUK8 injections.

EUK8 (Sigma-Aldrich, SML0742-50MG) was dissolved in sterile water at 5 mg/mL and injected intraperitoneally daily at 25 mg/kg body weight 1 d prior to infection and each day following infection until death or euthanasia.

#### MitoCocktail injections.

The MitoCocktail was formulated by combining Alpha-Lipoic acid (300 mg Capsules, SWU190), Coenzyme Q10 (200 mg Capsules, SWU035), L-Arginine (850 mg Capsules, SW1713), Vitamin-B1/Thiamin (100 mg Capsules, SW016), Vitamin-B2/Riboflavin (100 mg Capsules, SW018), Vitamin-B7/Biotin (5,000 μg Capsules, SW877), Vitamin-B9/Folic Acid (800 μg Capsules, SW035) purchased from Swanson®—Vitamins; L-Carnitine (500 mg Capsules, #01532), Creatin (1,000 mg Capsules, #01529), Vitamin-B3/Niacin (#00372, 500 mg Capsules) from Life Extension®; and Vitamin-C (500 mg Capsules) from Nature's Bounty® with KE4 Pro Ketone Ester Drink from KetoneAid® to supplement D-Beta Hydroxybutyrate (D-BHB). The daily intraperitoneally injections of our MitoCocktail included Alpha-Lipoic acid 4 mg/kg, Coenzyme Q10 2.7 mg/kg, L-Arginine 11.3 mg/kg, Vitamin-B1/Thiamin 1.3 mg/kg, Vitamin-B2/Riboflavin 1.3 mg/kg, Vitamin-B7/Biotin 66.7 μg/kg, Vitamin-B9/Folic Acid 10.7 μg/kg, L-Carnitine 6.7 mg/kg, Creatin 13.3 mg/kg, Vitamin-B3/Niacin 6.7 mg/kg, Vitamin-C 6.7 mg/kg, D-BHB 38.5 mg/kg body weight.

#### EUK8 plus MitoCocktail infecitons.

For injections including EUK8 and MitoCocktail, EUK8 was added to the MitoCocktail so that the final injectable concentration of EUK8 was at 25 mg/kg body weight.

#### Infection models and sample collection.

Mice were housed in the University of Pennsylvania ABSL3 facility on a 12:12 light cycle using autoclaved cages (Tecniplast, EM500), irradiated Bed-o-Cob (ScottPharma, Bed-o-Cob 4RB), ad libitum irradiated chow (LabDiet, PicoLab Select Rodent 50 IF/6F 5V5R), and autoclaved water bottles. Twelve-week-old mice were infected following light sedation, using 50 mg/kg ketamine and 15 mg/kg xylazine, by intranasal inoculation with 2.5 × 10^5^ or 1.0 × 10^5^ PFU of the SARS-CoV-2 of WA1, or 5.0 × 10^5^ PFU of the SARS-CoV-2 Omicron variant (BA.1) diluted in 40 μL DMEM or DMEM alone (mock-infection).

Blinded groups for mice were used throughout the study to limit investigator subjectivity. The extent of pathology was scored [scored 1 to 5, 1 = healthy, 2 = lethargic/reduced mobility, 3 = change in respiration (increased or decreased), 4 = labored breathing, 5 = moribund/dead] 1 d before and each day following infection. Mice were euthanized by an overdose of isoflurane anesthetic and samples were collected at select time points or upon becoming moribund (≥20% weight loss). Samples collected included lungs, BALF, and serum. Viral inactivation in BALF and serum was achieved by heat inactivation at 55 °C for 1 h and viral inactivation in the lung was performed by drop-fixing the left lobe in 10% natural buffered formalin and the right lung in TRIzol™ Reagent (Invitrogen, 15596026). For treatment experiments, mice were injected intraperitoneally daily with sterile water, EUK8, MitoCocktail, or MitoCocktail plus EUK8 1 d before infection and each day following infection until mice were collected.

### Quantification of Levels of Viral Transcripts and Cell-Free mtDNA.

#### Assessment of viral transcripts from mouse lungs.

RNA was collected from mouse lungs using TRIzol™ Reagent (Invitrogen, 15596026). Lung RNA was quantified using Nanodrop and reverse transcribed using the SuperScript™ IV First-Strand Synthesis System (Thermo Fisher Scientific, 18091200). Levels of SARS-CoV-2 transcripts were determined via TaqMan Real-Time PCR (RT-PCR) assays to detect the viral N protein mRNA (Thermo Scientific, Vi07918637_s1) and ORF1ab mRNA (Thermo Scientific, Vi07921935_s1), compared to 18S ribosomal RNA (Thermo Scientific, Mm03928990_g1).

#### Detection of mtDNA from serum.

mtDNA levels were assessed from cell lysates centrifuged for 15 min at 1,000 g. Protein concentrations were determined by Bradford assay (Bio-Rad protein assay, Bio-Rad, Hercules, CA). DNA was isolated from equal amounts of protein using the DNA Clean & Concentrator-5 (Zymo Research, 11-303) and mtDNA levels quantified via TaqMan RT-PCR of the mtDNA *MT-ND5* using the TaqMan probe (Thermo Scientific, Mm04225325_g1).

### Quantification of HIF-1α, INFβ1, and IL-1β Protein Levels via ELISA.

Protein concentrations from BALF and lung samples were quantified from TRIzol™ Reagent (Invitrogen, 15596026) using the Bio-Rad protein assay (Bio-Rad, Hercules, CA). Mouse HIF-1α was assayed utilizing the Human/Mouse Total HIF-1 alpha/HIF-1 alpha DuoSet IC enzyme-linked immunosorbent assay (ELISA) (R&D Systems, DYC1935-2); IFNβ using the Mouse IFN beta ELISA Kit (Abcam, ab252363); and IL1β utilizing the Mouse IL-1 beta ELISA Kit (Abcam, ab197742).

### Histological, IHC, TUNEL Staining Analysis.

#### Histological analysis.

Lungs from mice were collected, fixed in formalin, embedded in paraffin, sectioned, placed on glass slides, stained with Hematoxylin and Eosin (H&E), and evaluated in a blinded manner by a board-certified veterinary pathologist. Microscopic findings were quantified by severity score: 1 = minimal (rare foci with little tissue damage affecting less than 10% of the section), 2 = mild (multiple foci with tissue damage affecting less than 20% of the section), 3 = moderate (multiple more extensive foci with tissue damage affecting less than 50% of the section), 4 = marked (50 to 80% of tissue affected), or 5 = severe (greater than 80% of tissue affected).

#### IHC processing and image analysis.

Paraffin-embedded samples were sectioned at 4 μm, and IHC was performed using a Bond Rx autostainer (Leica Biosystems). Primary antibodies were HIF-1α (HIF-1 alpha Antibody, Novus Biologicals, H1alpha67 [NB100-105]); human catalase (Abcam, ab76024 [EP1929Y]); SARS-CoV-2 2-N (Nucleocapsid Antibody, GeneTex, GTX635686 [HL448]) (1:1,000). Secondary antibody was rabbit anti-rat secondary (Vector, 1:100) and staining enhanced using Bond Polymer Refine Detection (Leica Biosystems). After staining, sections were dehydrated and film cover slipped using a TissueTek-Prisma and Coverslipper (Sakura). Whole slide scanning (40×) was performed using an Aperio AT2 (Leica Biosystems). HALO image analysis (Indica Labs) was performed on whole slide images to quantify cell number and size, percentage of stained cells, and staining intensity.

### RNA Library Sequencing and Transcript Analysis.

RNA was assessed using the NanoDrop spectrophotometer (ThermoFisher Scientific) and quantified using the Qubit Flex (Thermofisher Scientific) and Qubit RNA HS Assay Kit (Thermofisher Scientific, Q32855). The RNA was diluted to 1.43 ng/μL and reverse transcribed into cDNA using the Ion Torrent™ NGS Reverse Transcription Kit (Cat. No. A45003). The RNA library was prepared using the Ion Torrent S5 Chef and Ion AmpliSeq Kit for Chef DL8 (Thermofisher Scientific, A29024) and sequenced using the Ion Torrent S5 Sequencer and Ion 540 Kit (Thermofisher Scientific, A30011). RNAseq reads were aligned to the *Mus musculus* C57Bl/6 genome (v1.100) and raw read counts calculated using the Torrent Server 5.16.1 Data Management Auto Actions Tool with the ampliSeqRNA and CoverageAnalysis analysis modules. Raw read counts were normalized in R (version 4.2.2) using the “DESeq2” (version 1.38.3) package. Also in R, volcano plots were generated using the “EnhancedVolcano” (version 1.16.0) package, and Heatmaps were generated using the “ComplexHeatmap”(29) (version 2.15.1), and “ggplot2” (version 3.4.1) packages. Gene Set Enrichment Analysis (GSEA) was performed using GSEA 4.3.2. Pathway analysis in GSEA was done utilizing custom-made Gene Set files (1) and (39). GO data were generated using “clusterProfiler” (version 4.6.2), “AnnotationDbi” (version 1.60.1), and “org.Mm.eg.db” (version 3.16.0) packages in R. GO and GSEA plots were generated in GraphPad Prism 9.

### Lung mROS Production Assessed by MDA Assay.

Twelve-week-old hACE2-mCAT mice were injected intraperitoneally with 5 mg/kg of lipopolysaccharide (LPS) (Sigma-Aldrich, L2630) or as a control with distilled water (dH_2_O) (hACE2-mCAT + dH_2_O). Additional 12-wk-old mice were injected intraperitoneally with either 25 mg/kg EUK8 (hACE2 + EUK8) or dH_2_O (hACE2 + dH_2_O) and the next day injected with 5 mg/kg of LPS. Eighteen hours after LPS treatment, mice were euthanized by cervical dislocation, and the lungs were removed and placed on ice. Lungs were washed with the isolation buffer [215 mM mannitol, 75 mM sucrose, 0.1% bovine serum albumin, 1 mM ethylene glycol-bis(β-aminoethyl ether)-N,N,N',N'-tetraacetic acid (EGTA), and 20 mM 4-(2-hydroxyethyl)-1-piperazineethanesulfonic acid (HEPES) (Na^+^) (pH 7.2)] containing protease inhibitor (Sigma-Aldrich, 4693116001) and then homogenized using an Omni Tissue Homogenizer. Whole lung homogenates were clarified at 20,000 g for 10 min, and protein amounts were determined by Bradford assay (Bio-Rad protein assay, Bio-Rad, Hercules, CA). One hundred micrograms of isolated lung homogenate were assayed using the Lipid Peroxidation (MDA) Assay Kit (Abcam, ab233471), per the manufacturer's instructions.

### Statistical and Reproducibility.

One-way ANOVA was performed on data from three or more groups, followed by a post hoc Tukey's Honestly Significant Difference (HSD) test of statistical differences. When comparing between two groups, an unpaired two-tail Student's *t* test was used. All statistical analysis was done on GraphPad Prism 9.01. For student's *t* test *P < value 0.05, **P < value 0.01, ***P < value 0.001, ****P < value 0.0001).

## Supplementary Material

Appendix 01 (PDF)

## Data Availability

The published article includes all datasets generated and analyzed during this study. The RNA-seq data are deposited on the Gene Expression Omnibus (GEO) repository (40). This study did not generate new unique reagents.

## References

[1] J. W. Guarnieri , Core mitochondrial genes are down-regulated during SARS-CoV-2 infection of rodent and human hosts. Sci. Transl. Med. **15**, eabq1533 (2023). 37556555 10.1126/scitranslmed.abq1533 PMC11624572

[2] J. W. Guarnieri , SARS-COV-2 viroporins activate the NLRP3-inflammasome by the mitochondrial permeability transition pore. Front. Immunol. **14**, 1064293 (2023). 36891303 10.3389/fimmu.2023.1064293 PMC9986324

[3] M. Cortese , Integrative imaging reveals SARS-CoV-2-induced reshaping of subcellular morphologies. Cell Host Microbe. **28**, 853–866 e855 (2020). 33245857 10.1016/j.chom.2020.11.003 PMC7670925

[4] P. Wang , A cross-talk between epithelium and endothelium mediates human alveolar-capillary injury during SARS-CoV-2 infection. Cell Death Dis. **11**, 1042 (2020). 33293527 10.1038/s41419-020-03252-9 PMC7721862

[5] A. C. Codo , Elevated glucose levels favor SARS-CoV-2 infection and monocyte response through a HIF-1α/glycolysis-dependent axis. Cell Metab. **32**, 437–446.e5 (2020). 32697943 10.1016/j.cmet.2020.07.007 PMC7367032

[6] I. Papandreou, R. A. Cairns, L. Fontana, A. L. Lim, N. C. Denko, HIF-1 mediates adaptation to hypoxia by actively downregulating mitochondrial oxygen consumption. Cell Metab. **3**, 187–197 (2006). 16517406 10.1016/j.cmet.2006.01.012

[7] H. Zhang , HIF-1 inhibits mitochondrial biogenesis and cellular respiration in VHL-deficient renal cell carcinoma by repression of C-MYC activity. Cancer Cell **11**, 407–420 (2007). 17482131 10.1016/j.ccr.2007.04.001

[8] D. Bojkova , Proteomics of SARS-CoV-2-infected host cells reveals therapy targets. Nature **583**, 469–472 (2020). 32408336 10.1038/s41586-020-2332-7 PMC7616921

[9] X. Duan , An airway organoid-based screen identifies a role for the HIF1alpha-glycolysis axis in SARS-CoV-2 infection. Cell Rep. **37**, 109920 (2021). 34731648 10.1016/j.celrep.2021.109920 PMC8516798

[10] B. E. Nilsson-Payant , The NF-kappaB transcriptional footprint is essential for SARS-CoV-2 replication. J. Virol. **95**, e0125721 (2021). 34523966 10.1128/JVI.01257-21 PMC8577386

[11] S. Wang , A single-cell transcriptomic landscape of the lungs of patients with COVID-19. Nat. Cell Biol. **23**, 1314–1328 (2021). 34876692 10.1038/s41556-021-00796-6 PMC8650955

[12] B. Zhu , Inhibition of the mitochondrial pyruvate carrier simultaneously mitigates hyperinflammation and hyperglycemia in COVID-19. Sci. Immunol. **8**, eadf0348 (2023). 36821695 10.1126/sciimmunol.adf0348 PMC9972900

[13] S. Ajaz , Mitochondrial metabolic manipulation by SARS-CoV-2 in peripheral blood mononuclear cells of patients with COVID-19. Am. J. Physiol. Cell Physiol. **320**, C57–C65 (2021). 33151090 10.1152/ajpcell.00426.2020 PMC7816428

[14] L. Gibellini , Altered bioenergetics and mitochondrial dysfunction of monocytes in patients with COVID-19 pneumonia. EMBO Mol. Med. **12**, e13001 (2020). 33078545 10.15252/emmm.202013001 PMC7645870

[15] R. Borella , Metabolic reprograming shapes neutrophil functions in severe COVID-19. Eur. J. Immunol. **52**, 484–502 (2022). 34870329 10.1002/eji.202149481

[16] H. Medini, A. Zirman, D. Mishmar, Immune system cells from COVID-19 patients display compromised mitochondrial-nuclear expression co-regulation and rewiring toward glycolysis. iScience **24**, 103471 (2021). 34812416 10.1016/j.isci.2021.103471 PMC8599136

[17] M. Tian , HIF-1alpha promotes SARS-CoV-2 infection and aggravates inflammatory responses to COVID-19. Signal Transduct. Target Ther. **6**, 308 (2021). 34408131 10.1038/s41392-021-00726-w PMC8371950

[18] Z. Zhong , New mitochondrial DNA synthesis enables NLRP3 inflammasome activation. Nature **560**, 198–203 (2018). 30046112 10.1038/s41586-018-0372-z PMC6329306

[19] A. P. West, G. S. Shadel, Mitochondrial DNA in innate immune responses and inflammatory pathology. Nat. Rev. Immunol. **17**, 363–375 (2017). 28393922 10.1038/nri.2017.21 PMC7289178

[20] T. Kawai, S. Akira, Signaling to NF-kappaB by Toll-like receptors. Trends Mol Med **13**, 460–469 (2007). 18029230 10.1016/j.molmed.2007.09.002

[21] T. J. Costa , Mitochondrial DNA and TLR9 activation contribute to SARS-CoV-2-induced endothelial cell damage. Vascul. Pharmacol. **142**, 106946 (2022). 34838735 10.1016/j.vph.2021.106946 PMC8612754

[22] S. Marchi, E. Guilbaud, S. W. G. Tait, T. Yamazaki, L. Galluzzi, Mitochondrial control of inflammation. Nat. Rev. Immunol. **23**, 159–173 (2023). 35879417 10.1038/s41577-022-00760-x PMC9310369

[23] J. Chu , Pharmacological inhibition of fatty acid synthesis blocks SARS-CoV-2 replication. Nat. Metab. **3**, 1466–1475 (2021). 34580494 10.1038/s42255-021-00479-4 PMC8475461

[24] S. Li , Metabolic reprogramming and epigenetic changes of vital organs in SARS-CoV-2-induced systemic toxicity. JCI insight **6**, e145027 (2021). 33284134 10.1172/jci.insight.145027 PMC7934846

[25] S. E. Schriner , Extension of murine life span by overexpression of catalase targeted to mitochondria. Science **308**, 1909–1911 (2005). 15879174 10.1126/science.1106653

[26] H. Y. Lee , Targeted expression of catalase to mitochondria prevents age-associated reductions in mitochondrial function and insulin resistance. Cell Metab. **12**, 668–674 (2010). 21109199 10.1016/j.cmet.2010.11.004 PMC3013349

[27] S. Melov , Lifespan extension and rescue of spongiform encephalopathy in superoxide dismutase 2 nullizygous mice treated with superoxide dismutase-catalase mimetics. J. Neurosci. **21**, 8348–8353 (2001). 11606622 10.1523/JNEUROSCI.21-21-08348.2001 PMC6762800

[28] J. W. Guarnieri , SARS-CoV-2 mitochondrial metabolic and epigenomic reprogramming in COVID-19. Pharmacol. Res. **204**, 107170 (2024). 38614374 10.1016/j.phrs.2024.107170

[29] Z. Gu, R. Eils, M. Schlesner, Complex heatmaps reveal patterns and correlations in multidimensional genomic data. Bioinformatics **32**, 2847–2849 (2016). 27207943 10.1093/bioinformatics/btw313

[30] J. Nassour , Telomere-to-mitochondria signalling by ZBP1 mediates replicative crisis. Nature **614**, 767–773 (2023). 36755096 10.1038/s41586-023-05710-8 PMC9946831

[31] R. Karki , ZBP1-dependent inflammatory cell death, PANoptosis, and cytokine storm disrupt IFN therapeutic efficacy during coronavirus infection. Sci. Immunol. **7**, eabo6294 (2022). 35587515 10.1126/sciimmunol.abo6294 PMC9161373

[32] Y. Li , Dilated cardiomyopathy and neonatal lethality in mutant mice lacking manganese superoxide dismutase. Nat. Genet. **11**, 376–381 (1995). 7493016 10.1038/ng1295-376

[33] K. J. Morten, B. A. Ackrell, S. Melov, Mitochondrial reactive oxygen species in mice lacking superoxide dismutase 2: Attenuation via antioxidant treatment. J. Biol. Chem. **281**, 3354–3359 (2006). 16326710 10.1074/jbc.M509261200

[34] S. Melov , Mitochondrial disease in superoxide dismutase 2 mutant mice. Proc. Natl. Acad. Sci. U.S.A. **96**, 846–851 (1999). 9927656 10.1073/pnas.96.3.846 PMC15313

[35] S. Parikh , A modern approach to the treatment of mitochondrial disease. Curr. Treat Options Neurol. **11**, 414–430 (2009). 19891905 10.1007/s11940-009-0046-0 PMC3561461

[36] I. Barcelos, E. Shadiack, R. D. Ganetzky, M. J. Falk, Mitochondrial medicine therapies: Rationale, evidence, and dosing guidelines. Curr. Opin. Pediatr. **32**, 707–718 (2020). 33105273 10.1097/MOP.0000000000000954 PMC7774245

[37] S. L. Weiss, D. Zhang, S. Farooqi, D. C. Wallace, Sodium butyrate reverses lipopolysaccharide-induced mitochondrial dysfunction in lymphoblasts. J. Cell. Mol. Med. **26**, 3290–3293 (2022). 35587004 10.1111/jcmm.17342 PMC9170810

[38] B. Miller , Host mitochondrial transcriptome response to SARS-CoV-2 in multiple cell models and clinical samples. Sci. Rep. **11**, 3 (2021). 33420163 10.1038/s41598-020-79552-z PMC7794290

[39] J. W. Guarnieri , Lethal COVID-19 associates with RAAS-induced inflammation for multiple organ damage including mediastinal lymph nodes. bioRxiv [Preprint] (2023). 10.1101/2023.10.08.561395 (Accessed 27 November 2023). PMC11626201 39602262

[40] J. W. Guarnieri, G. A. Widjaja, D. C. Wallace, Data from “Mitochondrial Antioxidants Abate SARS-CoV-2 Lethality in Mice.” GEO. https://www.ncbi.nlm.nih.gov/geo/query/acc.cgi?acc=GSE246312. Deposited 26 October 2023.

