# Supplementary material for: Mitochondrial antioxidants abate SARS-COV-2 pathology in mice
Source: Proc Natl Acad Sci U S A. 2024 Jul 15;121(30):e2321972121. doi: 10.1073/pnas.2321972121 (PMC11287122; doi:10.1073/pnas.2321972121)
Supplement: Supplementary file 1 — Appendix 01 (PDF) [file pnas.2321972121.sapp.pdf]

PNAS

Supporting Information for

Mitochondrial Antioxidants Abate SARS-CoV-2 Pathology in Mice.

Joseph W. Guarnieri[1], Timothy Lie[1,2], Yentli E. Soto Albrecht[1,2], Peter Hewins[2,3], Kellie A. Jurado[2,3], Gabrielle A. Widjaja[1], Yi Zhu[4], Meagan J. McManus[1,4], Todd J. Kilbaugh[4], Kelsey Keith[5], Prasanth Portluri[1], Deanne Taylor[1,5,6], Alessia Angelin[1], Deborah G. Murdock[1], Douglas C. Wallace[1,6]*

Douglas C. Wallace[1,6]*
Email: WallaceD1@chop.edu

This PDF file includes:

Figures S1 to S3

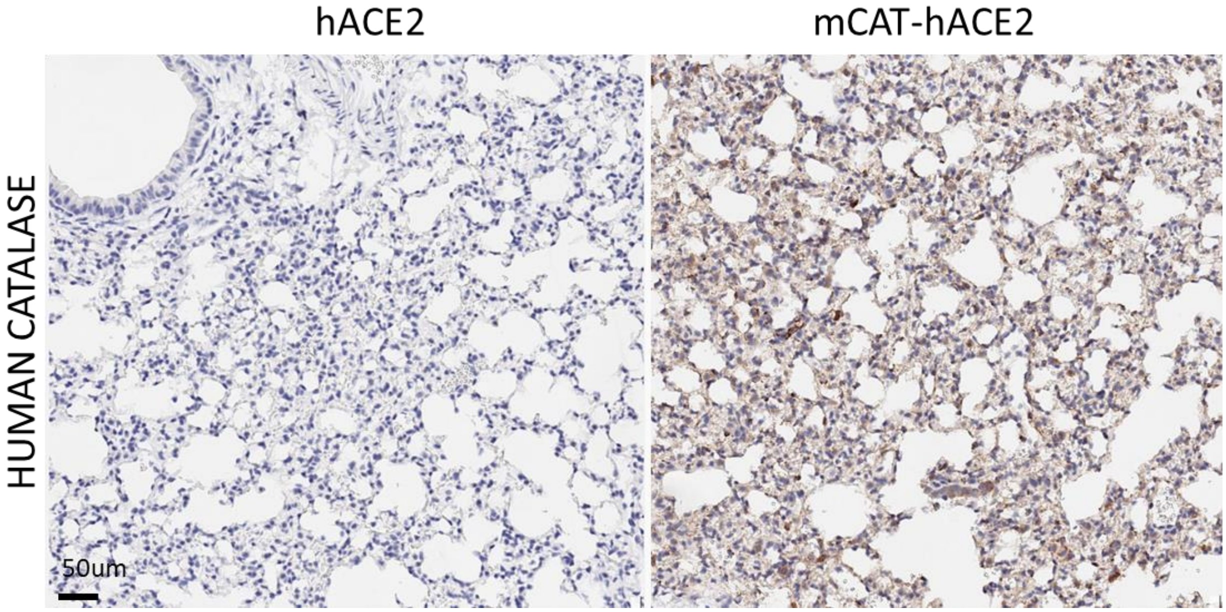

Fig. S1. Representative images of anti-human catalase antibody stained (brown) lungs with and without mCAT expression.

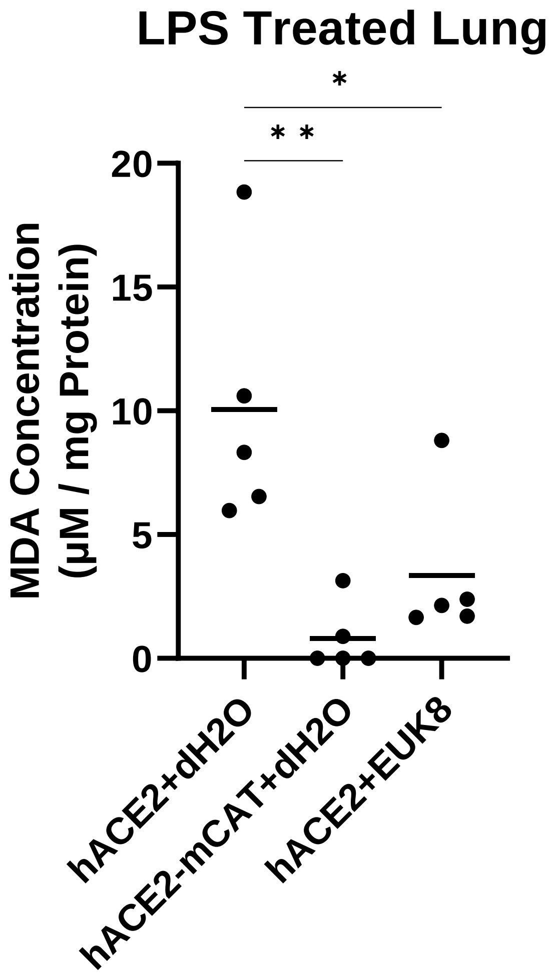

Figure S2. Lipid peroxidation (MDA) assay on lungs collected from 5 mg/kg LPS-treated (18hrs) mice with and without EUK8-treament (48hrs) or mCAT expression (n = 5 for each condition).

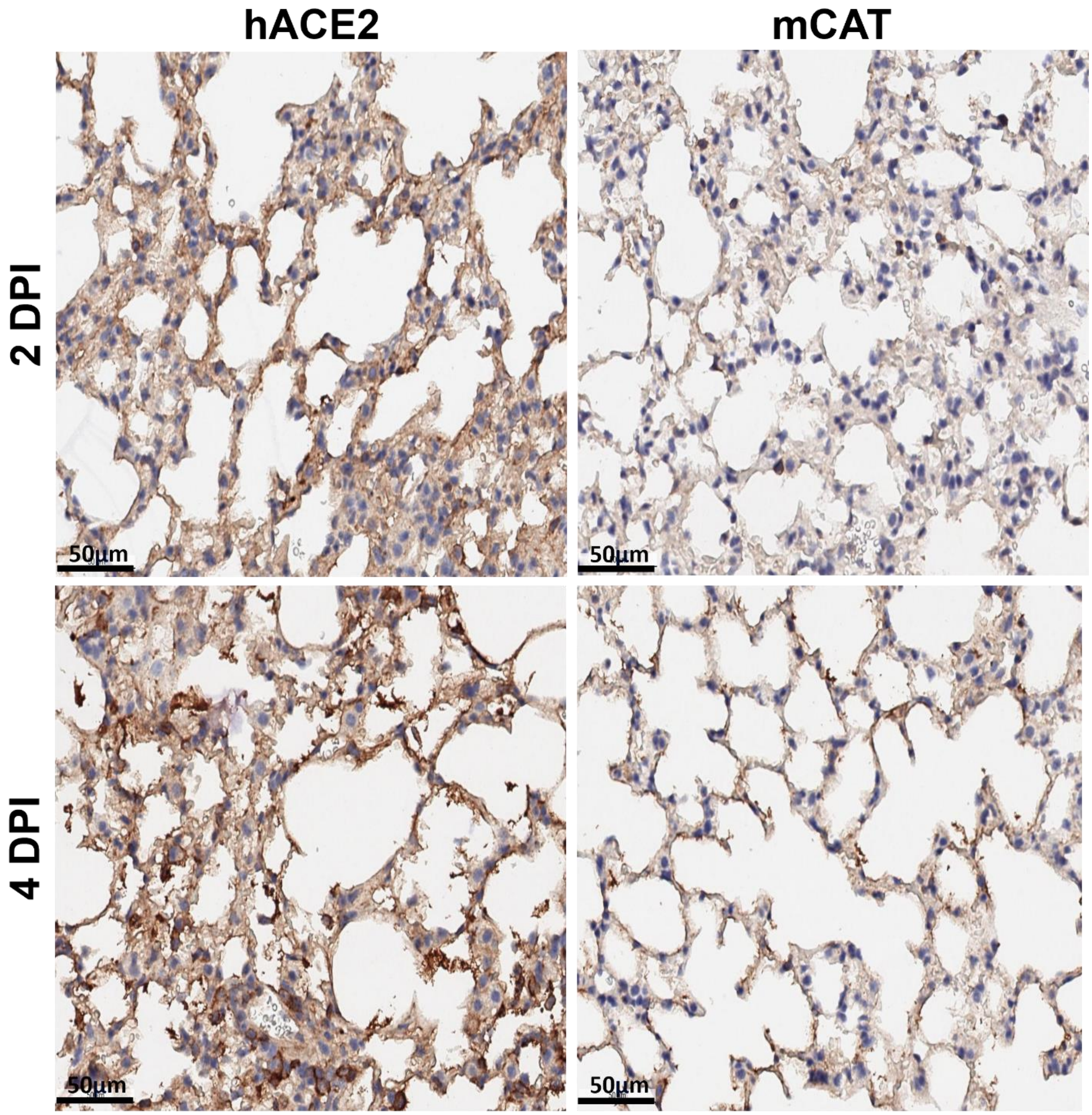

Figure S3. Representative images of anti-HIF-1α antibody stained (brown) infected mouse lungs with and without mCAT expression and analyzed at 2 and 4 DPI**,** quantification presented in **Fig. 1h.**
